# Palmitate and pyruvate carbon flux in response to choline and methionine in bovine neonatal hepatocytes

**DOI:** 10.1038/s41598-020-75956-z

**Published:** 2020-11-05

**Authors:** T. L. Chandler, S. J. Erb, William A. Myers, Pragney Deme, Norman J. Haughey, J. W. McFadden, H. M. White

**Affiliations:** 1 Department of Dairy Science, University of Wisconsin-Madison, Madison, WI 53706 USA; 2 Department of Animal Science, Cornell University, Ithaca, NY 14853 USA; 3 Division of Neuroimmunology and Neurological Infections, Department of Neurology, Johns Hopkins University School of Medicine, Baltimore, MD 21287 USA; 4 Present Address: Department of Population Medicine and Diagnostic Sciences, College of Veterinary Medicine, Cornell University, Ithaca, NY 14853 USA

**Keywords:** Lipidomics, Metabolism, Fat metabolism, Metabolic diseases

## Abstract

Choline and methionine may serve unique functions to alter hepatic energy metabolism. Our objective was to trace carbon flux through pathways of oxidation and glucose metabolism in bovine hepatocytes exposed to increasing concentrations of choline chloride (CC) and d,l-methionine (DLM). Primary hepatocytes were isolated from 4 Holstein calves and maintained for 24 h before treatment with CC (0, 10, 100, 1000 μmol/L) and DLM (0, 100, 300 μmol/L) in a factorial design. After 21 h, [1-^14^C]C16:0 or [2-^14^C]pyruvate was added to measure complete and incomplete oxidation, and cellular glycogen. Reactive oxygen species (ROS), cellular triglyceride (TG), and glucose and ß-hydroxybutyrate (BHB) export were quantified. Exported very-low density lipoprotein particles were isolated for untargeted lipidomics and to quantify TG. Interactions between CC and DLM, and contrasts for CC (0 vs. [10, 100, 1000 μmol/L] and linear and quadratic contrast 10, 100, 1000 μmol/L) and DLM (0 vs. [100, 300 μmol/L] and 100 vs. 300 μmol/L) were evaluated. Presence of CC increased complete oxidation of [1-^14^C]C16:0 and decreased BHB export. Glucose export was decreased, but cellular glycogen was increased by the presence of CC and increasing CC. Presence of CC decreased ROS and marginally decreased cellular TG. No interactions between CC and DLM were detected for these outcomes. These data suggest a hepato-protective role for CC to limit ROS and cellular TG accumulation, and to alter hepatic energy metabolism to support complete oxidation of FA and glycogen storage regardless of Met supply.

## Introduction

Homeorhetic changes to support the onset of lactation are conserved across mammalian species but are paramount in dairy cows given the extent of energy and glucose requirements to support milk production during the period of negative energy balance. Hepatic oxidation of fatty acid (FA) mobilized from adipose tissue lipolysis fuels gluconeogenesis which is required to provide glucose as a lactose precursor^1,2^. While FA provide a critical energy source postpartum, hepatic uptake of FA can exceed capacity for FA oxidation and triglyceride (TG) export as very low-density lipoproteins (VLDL) leading to lipidosis and increased ketogenesis^3^. Additionally, peripartum cows experience oxidative stress and inflammation^4^. Previous research in rodents^5,6^ and dairy cows^7–9^ suggests that methyl-donors choline and methionine (Met) can modulate lipid, glucose, and inflammatory pathways.

We hypothesized that choline and Met alter the flux of carbon through pathways of energy metabolism in the liver. Given the intersection of choline and Met metabolism^10^, our experiment was conducted as a factorial design to investigate the interaction between supply of choline and Met. The primary objective of this experiment was to investigate the effects of choline chloride (CC) and D,L-Met (DLM) on carbon flux of palmitate and pyruvate through pathways of oxidation and gluconeogenesis in primary bovine neonatal hepatocytes. In order to accomplish this objective, pathway flux was determined by tracing techniques, and production of labeled pathway products from palmitate and pyruvate were considered with quantification of other intermediates and products. Because choline and Met may serve unique functions to alter FA metabolism^10^ which may interact with their effects on the flux of carbon through pathways of energy metabolism^8^, palmitate and pyruvate metabolism were measured after hepatocytes were exposed to FA. Given the relevance of choline and Met to liver lipid metabolism, their effects on cellular TG, oxidation, and ketogenesis were examined. Markers of oxidative stress were investigated because of their association with oxidation and inflammation in the liver and responsiveness to choline and Met^11^. In addition, cellular glycogen, glucose export, and pyruvate metabolism were quantified to determine if choline or Met altered glucose metabolism. Additionally, VLDL particles were isolated from media to quantify TG and identify lipid species by untargeted lipidomics to determine if CC or DLM altered the lipid composition of VLDL particles, including phosphatidylcholine (PtdChol) and phosphatidylethanolamine (PtdEth).

## Results

### Fatty acid metabolism

#### Cellular and VLDL TG

**Figure 1:**
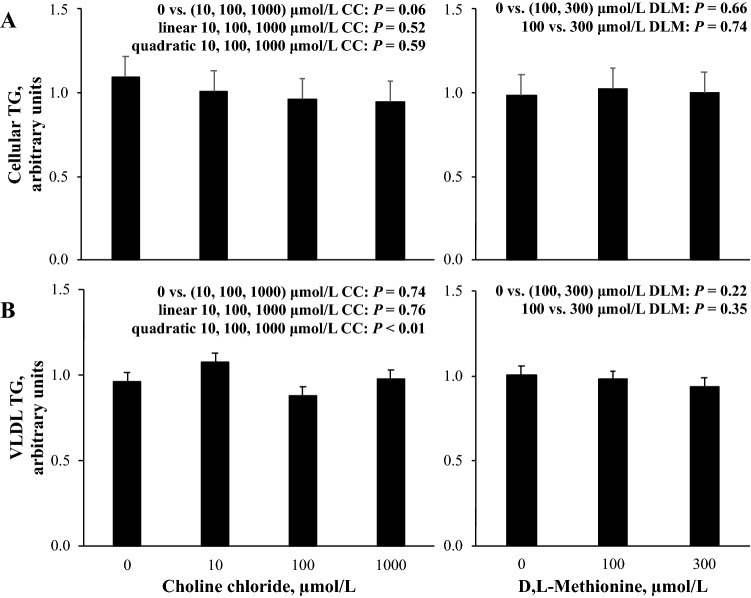
Cellular triglyceride (TG, **A**) in primary bovine neonatal hepatocytes and TG in very-low density lipoprotein (VLDL, **B**) exported from hepatocytes exposed to increasing concentrations of choline chloride (CC) and d,l-methionine (DLM) and a 1 mmol/L fatty acid (FA) cocktail for 24 h. Cellular and VLDL TG were quantified by colorimetric assay. Data were normalized to cellular DNA and expressed relative to the 0 μmol/L CC, 0 μmol/L DLM, 1 mmol/L FA treatment within each independent cell preparation. Values are least squares means, with SE represented by vertical bars. The *P*-values for contrast effects of CC and DLM are shown. Interactions between CC and DLM were not detected: *P* = 0.80 and *P* = 0.75 for (**A**, **B**), respectively. For the 0 μmol/L CC, 0 μmol/L DLM, 1 mmol/L FA treatment, three cell preparations averaged (± standard deviation) 21.9 ± 8.2 μmol/ug DNA of cellular TG and 58.5 ± 24.6 pmol/ug DNA of VLDL TG.

For the 0 μmol/L CC, 0 μmol/L DLM, 1 mmol/L FA treatment, three cell preparations averaged (± standard deviation) 21.9 ± 8.2 μmol/ug DNA of cellular TG and 58.5 ± 24.6 pmol/ug DNA of VLDL TG. The contrast effects of CC and DLM for cellular TG and VLDL TG quantified by colorimetric assay are presented in Fig. 1. There was a marginal effect for the presence of CC to decrease (*P* = 0.06) cellular TG, while no evidence (*P* > 0.10) for treatment effects of DLM were observed. The presence of CC or DLM did not appear to affect (*P* > 0.10) TG export in VLDL; however, increasing CC quadratically affected (*P* < 0.01) VLDL TG (Fig. 1). Cellular TG and VLDL TG quantified by colorimetric assay were not correlated (*r* = − 0.21, *P* = 0.22).

#### [1-^14^C]C16:0 oxidation

**Figure 2:**
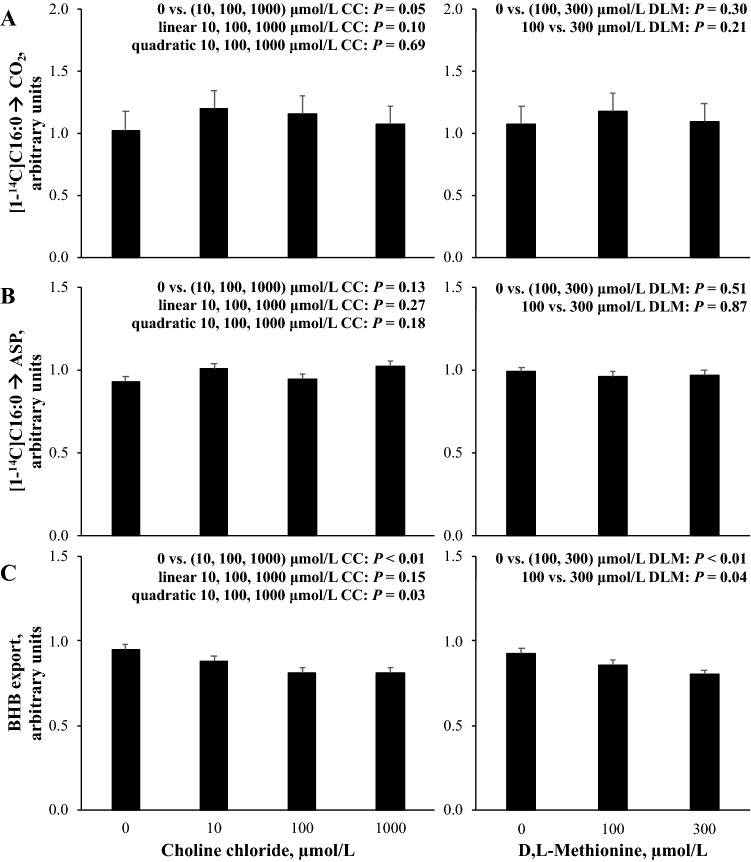
Relative rate of [1-^14^C]C16:0 oxidation to CO_2_ (**A**) or acid soluble products (ASP) (**B**), and BHB export (**C**) from primary bovine neonatal hepatocytes exposed to increasing concentrations of choline chloride (CC) and d,l-Methionine (DLM) and a 1 mmol/L fatty acid (FA) cocktail. Export of BHB was quantified after 24 h of treatment exposure and data were normalized to cellular DNA, and the average (± standard deviation) export of BHB for three cell preparations from the 0 μmol/L CC, 0 μmol/L DLM, 1 mmol/L FA treatment was 63.0 ± 21.2 nmol/µg DNA. Oxidation was quantified after 21 h of treatment exposure before a 3 h incubation with [1-^14^C]C16:0. The rate of [1-^14^C]C16:0 oxidation to CO_2_ was expressed as pmol ^14^C substrate metabolized to ^14^CO_2_·µg DNA^−1^·h^−1^ and the average (± standard deviation) rate for four cell preparations from the 0 μmol/L CC, 0 μmol/L DLM, 1 mmol/L FA treatment was 3.5 ± 0.9 pmol·µg DNA^−1^·h^−1^. The rate of [1-^14^C]C16:0 oxidation to ASP was expressed as pmol ^14^C substrate incorporated into ASP µg · DNA^−1^·h^−1^ and the average (± standard deviation) rate for four cell preparations from the 0 μmol/L CC, 0 μmol/L DLM, 1 mmol/L FA treatment was 3.0 ± 0.6 pmol·µg DNA^−1^·h^−1^. Data are expressed relative to the 0 μmol/L CC, 0 μmol/L DLM, 1 mmol/L FA treatment within each independent cell preparation. Values are least squares means, with SE represented by vertical bars. The *P-* values for contrast effects of CC and DLM are shown. Interactions between CC and DLM were not detected: *P* = 0.31, *P* = 0.27, and *P* = 0.27 for (**A**–**C**), respectively.

The contrast effects of CC and DLM on the metabolic rates of [1-^14^C]C16:0 metabolism and ß-hydroxybutyrate (BHB) export are presented in Fig. 2. The average (± standard deviation) export of BHB for three cell preparations from the 0 μmol/L CC, 0 μmol/L DLM, 1 mmol/L FA treatment was 63.0 ± 21.2 nmol/µg DNA. The rate of [1-^14^C]C16:0 oxidation to CO_2_ averaged (± standard deviation) 3.5 ± 0.9 pmol·µg DNA^−1^·h^−1^ from the 0 μmol/L CC, 0 μmol/L DLM, 1 mmol/L FA treatment for four cell preparations. The rate of [1-^14^C]C16:0 incomplete oxidation to acid-soluble products (ASP) averaged (± standard deviation) 3.0 ± 0.6 pmol·µg DNA^−1^·h^−1^ from the 0 μmol/L CC, 0 μmol/L DLM, 1 mmol/L FA treatment for four cell preparations. The relative rate of [1-^14^C]C16:0 complete oxidation to CO_2_ was increased (*P* = 0.05) by the presence of CC; however, increasing supply of CC resulted in a marginal (*P* = 0.10) linear decrease of [1-^14^C]C16:0 oxidation to CO_2_ (Fig. 2). Choline chloride did not appear to affect (*P* > 0.10) the relative rate of [1-^14^C]C16:0 incomplete oxidation to ASP. Oxidation of [1-^14^C]C16:0 to either CO_2_ or ASP did not appear to be affected (*P* > 0.10) by DLM. The export of BHB was decreased (*P* < 0.01) by the presence of CC and quadratically affected (*P* < 0.01) by increasing CC supply. The presence of DLM and increasing DLM from 100 to 300 µmol/L decreased (*P* < 0.01) BHB export.

#### Lipidomics

Table 1 presents the total number of lipid species classes identified in VLDL and partial Pearson correlations between total lipid species within class and cellular TG. Untargeted lipidomics revealed 168 TG, 28 diacylglycerol (DG), 32 PtdChol, 19 PtdEth, 18 phosphatidylserine (PS), 8 phosphatidylglycerol (PG), 12 lyso-phospholipid (Lyso-P), 10 sphingomyelin (SM), and 2 ceramide (Cer) species, as well as a cholesteryl ester (CE, 18:3) (Table 1). Lyso-phospholipids included lyso-phosphatidic acid, lyso-phosphatidylcholine, lyso-phosphatidylethanolamine, lyso-phosphatidylglycerol, and lyso-phosphatidylserine species. Cellular TG were not correlated to total TG species identified in VLDL by lipidomic analysis (*r* = 0.04, *P* = 0.81); however, cellular TG were negatively correlated to total PtdChol species (*r* = − 0.36, *P* = 0.04) and positively correlated to LPC (18:0) (*r* = 0.55, *P* < 0.01).

**Table 1:** Change in total lipid species detected by untargeted lipidomics in very-low density lipoprotein particles exported from primary bovine neonatal hepatocytes in response to choline chloride (CC) and D,L-Met (DLM)^1^.

Lipid class^1^	Species	*r*^2^	CC				DLM			SE	Contrast *P* value^3^				
			0	10	100	1000	0	100	300		1	2	3	4	5
TG	168	0.04	1.01	0.97	0.99	0.88	0.95	0.97	0.97	0.05		**			
DG	28	− 0.28	0.95	1.00	0.91	0.94	0.96	0.98	0.91	0.03			**		*
PtdChol	32	− 0.36**	0.92	0.94	0.96	0.90	0.91	0.94	0.95	0.06					
PtdEth	19	− 0.17	0.98	1.02	0.96	0.94	0.98	0.94	1.00	0.06					
PS	18	0.09	0.90	1.22	0.89	1.05	1.10	0.93	1.01	0.09			**		
PG	8	− 0.15	0.94	1.06	0.97	0.99	0.98	0.97	1.03	0.10					
Lyso-P	12	− 0.22	0.94	1.06	0.83	0.95	0.97	0.91	0.95	0.08			**		
LPC (18:0)		0.55**	1.00	1.21	0.82	0.69	0.96	0.89	0.94	0.10		**	**		
SM	10	− 0.21	0.96	0.97	0.98	0.93	0.97	0.96	0.94	0.02					
Cer	2	0.16	0.96	1.01	0.95	0.99	1.03	0.97	0.93	0.16					
CE (18:3)^4^	1	0.23	0.86	0.71	0.74	0.64	0.76	0.75	0.69	0.06	**				

^1^Triglyceride (TG), diacylglycerol (DG), phosphatidylcholine (PtdChol), phosphatidylethanolamine (PtdEth), phosphatidylserine (PS), phosphatidyl-glycerol (PG), lysophospholipid (Lyso-P), lysophosphatidylcholine (LPC), sphingomyelin (SM), ceramide (Cer), cholesteryl ester (CE).

^2^Partial correlation between respective lipids and cellular TG, **P < 0.05.

^3^Contrasts statements of (1) 0 μmol/L CC versus (10, 100, 1000 μmol/L CC); (2) linear 10, 100, 1000 μmol/L CC; (3) quadratic 10, 100, 1000 μmol/L CC; (4) 0 μmol/L DLM versus (100, 300 μmol/L DLM); (5) 100 μmol/L DLM versus 300 μmol/L DLM. *P < 0.10, **P < 0.05.

^4^Significant interaction between CC and DLM, *P* = 0.007.

**Figure 3:**
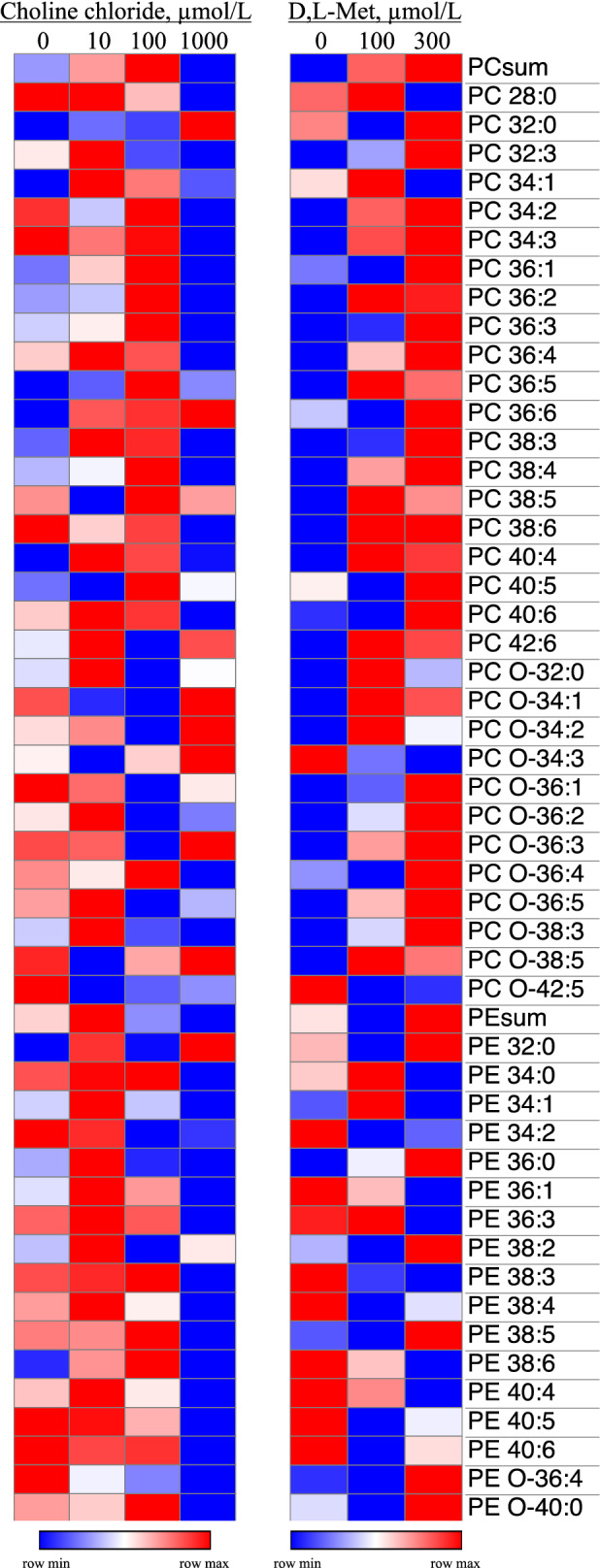
Lipidome profile of very-low density lipoprotein phosphatidylcholine (PtdChol) and phosphatidylethanolamine (PE). The heat map represents relative log values of the data normalized to cellular DNA. All PtdChol and PtdEth species identified are shown. No significant effects of CC or DLM were detected after correcting for False Discovery Rate (*P* < 0.10). Blue represents row minimum and red row maximum.

Contrast effects of CC and DLM on total lipid species, as well as lyso-phosphatidylcholine (LPC, 18:0) are presented in Table 1. Total TG, DG, PS, Lyso-P, and CE species were either linearly or quadratically (*P* < 0.10) affected by CC, while total DG was marginally decreased (*P* = 0.08) by increasing DLM from 100 to 300 µmol (Table 1). Lyso-phosphatidylcholine (18:0) was linearly (*P* = 0.02) and quadratically affected (*P* = 0.03) by increasing CC. A heat map representing the relative log values for CC and DLM treatments for all PtdChol and PtdEth species identified is presented in Fig. 3. No significant contrast effects for CC and DLM were detected after correcting for a 10% False Discovery Rate (FDR). A CC × DLM interaction was observed for CE (18:3) found in VLDL (*P* = 0.007, Table 1).

### Oxidative stress

**Figure 4:**
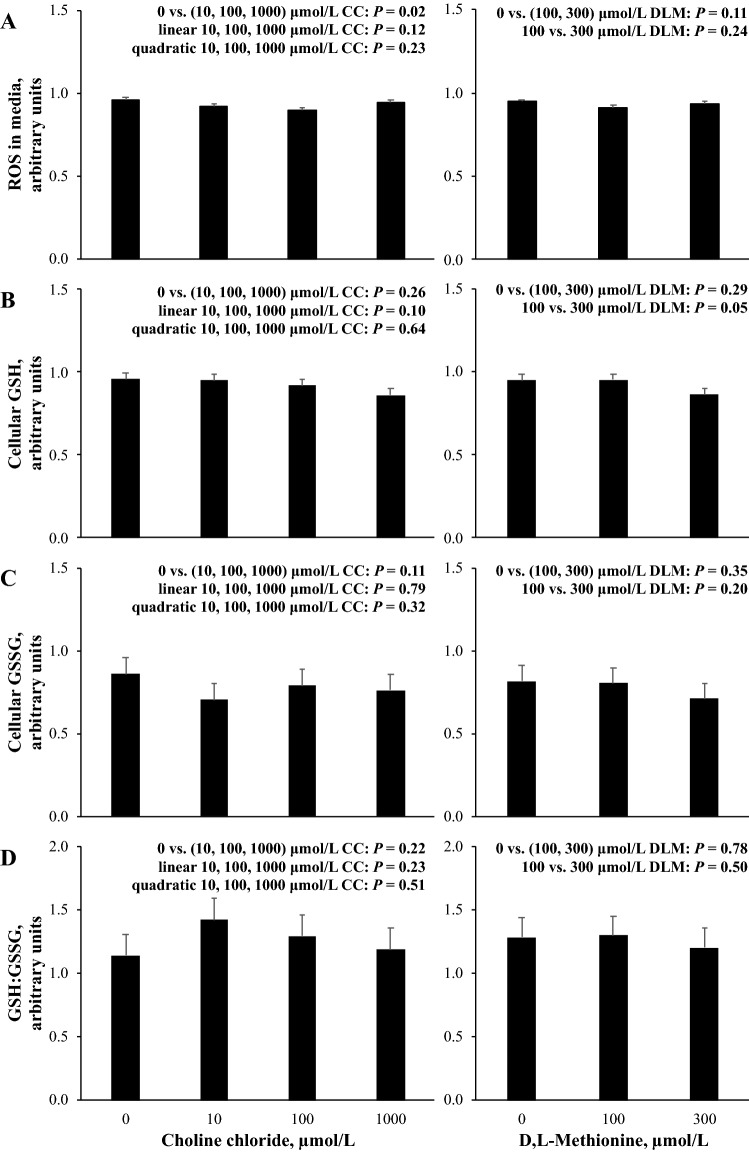
Relative concentration of reactive oxygen species (ROS) in media (**A**), cellular glutathione (GSH) (**B**), cellular glutathione disulfide (GSSG) (**C**), and the ratio of GSH:GSSG (**D**) in primary bovine neonatal hepatocytes exposed to increasing concentrations of choline chloride (CC) and d,l-Methionine (DLM) and a 1 mmol/L fatty acid (FA) cocktail for 24 h. Concentrations of ROS, GSH, GSSG, and GSH:GSSG are expressed relative to the 0 μmol/L DLM, 0 μmol/L CC, 1 mmol/L FA treatment within each independent cell preparation. Values are least squares means, with SE represented by vertical bars. The *P-*values for contrast effects of CC and DLM are shown. Interactions between CC and DLM were not detected: *P* = 0.23, *P* = 0.18, *P* = 0.41, and *P* = 0.83 for (**A**–**D**), respectively.

The contrast effects of CC and DLM on reactive oxygen species (ROS) in media, cellular glutathione (GSH) and glutathione disulfide (GSSG), and the ratio of GSH:GSSG are presented in Fig. 4. The accumulation of ROS in cell culture media was decreased (*P* < 0.05) by the presence of CC, but there was no evidence for treatment differences (*P* > 0.10) with the presence of DLM or by increasing DLM supply. Increasing supply of CC resulted in a marginal linear decrease (*P* = 0.10) in GSH, and GSH was decreased (*P* = 0.05) by increasing DLM from 100 to 300 µmol/L; however, no evidence for treatment differences (*P* > 0.10) by CC and DLM on cellular GSSG and the ratio of GSH:GSSG were observed.

### Glucose metabolism

**Figure 5:**
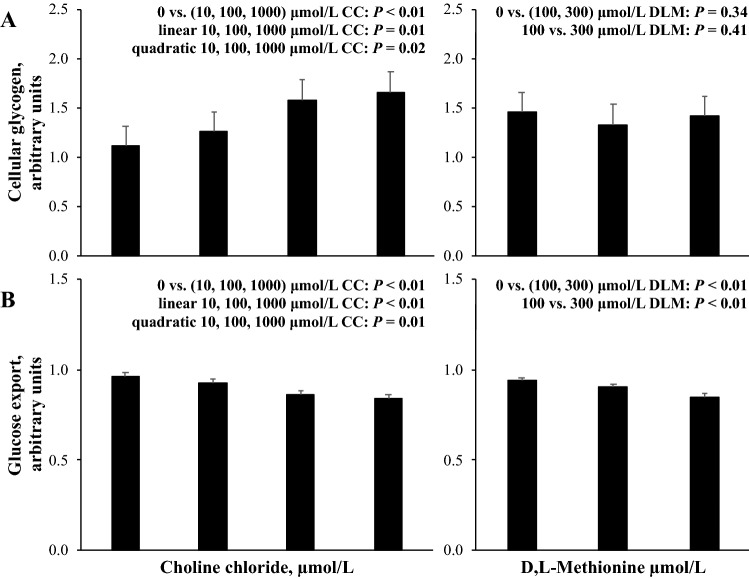
Cellular glycogen (**A**) and glucose export (**B**) from primary bovine neonatal hepatocytes exposed to increasing concentrations of choline chloride (CC) and d,l-Methionine (DLM) and a 1 mmol/L fatty acid (FA) cocktail for 24 h. Export of glucose was quantified after 24 h of treatment exposure and data were normalized to cellular DNA, and the average (± standard deviation) export of glucose for three cell preparations from the 0 μmol/L CC, 0 μmol/L DLM, 1 mmol/L FA treatment over 24 h was 112 ± 44 nmol/µg DNA. Cellular glycogen was quantified after 21 h of treatment exposure and a 3 h incubation with [2-^14^C] sodium pyruvate, and for four cell preparations glucose in cellular glycogen averaged (± standard deviation) 12.2 ± 2.9 nmol/µg DNA. Data are expressed relative to the 0 μmol/L DLM, 0 μmol/L CC, 1 mmol/L FA treatment within each independent cell preparation. Values are least squares means, with SE represented by vertical bars. The *P-*values for contrast effects of CC and DLM are shown. Interactions between CC and DLM were not detected: *P* = 0.21 and *P* = 0.42, for (**A**, **B**), respectively.

Glucose in cellular glycogen averaged (± standard deviation) 12.2 ± 2.9 nmol/µg DNA in the 0 μmol/L CC, 0 μmol/L DLM, 1 mmol/L FA treatment in four cell preparations, and export of glucose into culture media for three cell preparations from the 0 μmol/L CC, 0 μmol/L DLM, 1 mmol/L FA treatment over 24 h was 112 ± 44 nmol/µg DNA. The effects of CC and DLM on cellular glycogen and glucose export are presented in Fig. 5. Cellular glycogen was increased (*P* < 0.01) by the presence of CC, and linearly increased (*P* = 0.01) and quadratically affected (*P* = 0.02) by increasing supply of CC but no evidence for treatment differences (*P* > 0.10) by DLM were observed. Both the presence and increasing supply of CC linearly decreased (*P* < 0.01) glucose export and increasing CC supply quadratically affected (*P* = 0.01) glucose export. Similar to CC, the presence of DLM and increasing DLM from 100 to 300 µmol/L decreased (*P* < 0.01) glucose export.

**Figure 6:**
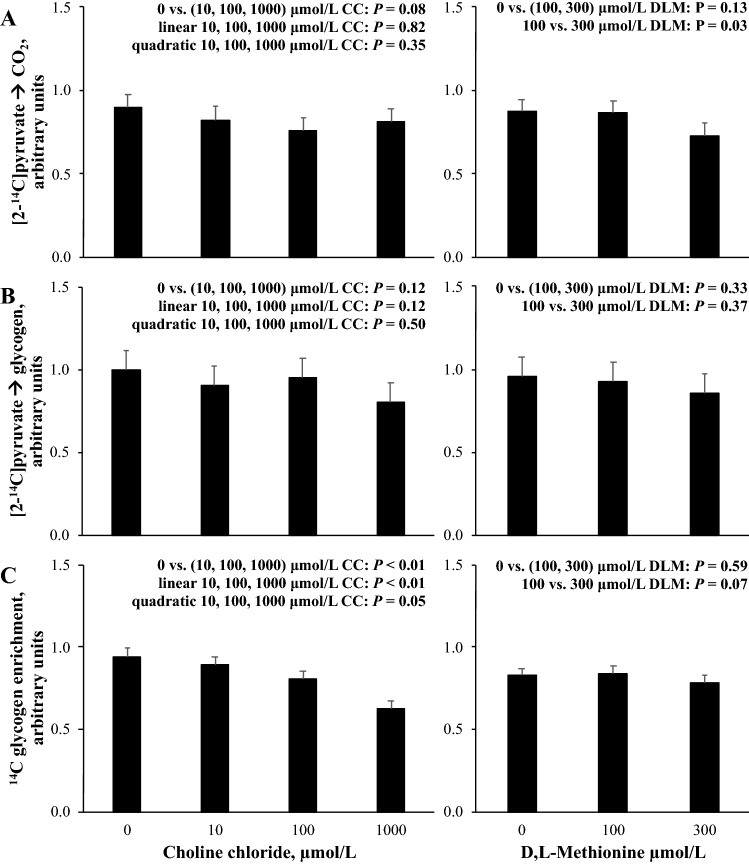
Relative rate of [2-^14^C] sodium pyruvate oxidation to CO_2_ (**A**), incorporation of [2-^14^C] sodium pyruvate into glycogen (**B**), and glycogen enrichment with ^14^C from [2-^14^C] sodium pyruvate (**C**) in primary bovine neonatal hepatocytes exposed to increasing concentrations of choline chloride (CC) and d,l-Methionine (DLM) and a 1 mmol/L fatty acid (FA) cocktail for 21 h before a 3 h incubation with [2-^14^C] sodium pyruvate. The rate of [2-^14^C] sodium pyruvate oxidation to CO_2_ was expressed as pmol ^14^C substrate metabolized to ^14^CO_2_·µg DNA^−1^·h^−1^, and the average (± standard deviation) rate for four cell preparations from the 0 μmol/L CC, 0 μmol/L DLM, 1 mmol/L FA treatment was 8.8 ± 1.2 pmol·µg DNA^−1^·h^−1^. The rate of [2-^14^C] sodium pyruvate incorporation into glycogen was expressed as pmol ^14^C substrate recovered in glucose·µg DNA^−1^·h^−1^, and the average (± standard deviation) rate for four cell preparations from the 0 μmol/L CC, 0 μmol/L DLM, 1 mmol/L FA treatment was 8.1 ± 4.0 pmol·µg DNA^−1^·h^−1^. Glycogen enrichment with ^14^C from [2-^14^C] sodium pyruvate was expressed as pmol of ^14^C substrate per pmol of glucose in glycogen, and the average (± standard deviation) enrichment for four cell preparations from the 0 μmol/L CC, 0 μmol/L DLM, 1 mmol/L FA treatment was 0.19 ± 0.07 pmol per pmol of glucose in glycogen. Data are expressed relative to the 0 μmol/L CC, 0 μmol/L DLM, 1 mmol/L FA treatment within each independent cell preparation. Values are least squares means, with SE represented by vertical bars. The *P-*values for contrast effects of CC and DLM are shown. Interactions between CC and DLM were not detected: *P* = 0.95, *P* = 0.95, and *P* = 0.37 for (**A**–**C**), respectively.

The metabolic rate of [2-^14^C] sodium pyruvate metabolism in response to CC and DLM is presented in Fig. 6. For the four cell preparations, the rate of [2-^14^C] sodium pyruvate oxidation to CO_2_ averaged (± standard deviation) 8.8 ± 1.2 pmol·µg DNA^−1^·h^−1^ from the 0 μmol/L CC, 0 μmol/L DLM, 1 mmol/L FA treatment, and the rate of [2-^14^C] sodium pyruvate incorporation into glycogen averaged (± standard deviation) 8.2 ± 4.0 pmol·µg DNA^−1^·h^−1^ from the 0 μmol/L CC, 0 μmol/L DLM, 1 mmol/L FA treatment. Glycogen enrichment with ^14^C from [2-^14^C] sodium pyruvate averaged (± standard deviation) 0.19 ± 0.07 pmol per pmol of glucose in glycogen from the 0 μmol/L CC, 0 μmol/L DLM, 1 mmol/L FA treatment. Oxidation of [2-^14^C] sodium pyruvate to CO_2_ was marginally decreased (*P* = 0.08) by the presence of CC and decreased (*P* < 0.05) when DLM was increased from 100 to 300 µmol/L (Fig. 6). The rate of [2-^14^C] sodium pyruvate incorporation into glycogen did not appear to be affected (*P* > 0.10) by either CC or DLM. Enrichment of glycogen with ^14^C from [2-^14^C] sodium pyruvate was decreased (*P* < 0.01) by the presence of CC, and linearly decreased (*P* < 0.01) and quadratically affected (*P* = 0.05) by increasing CC supply. Increasing DLM from 100 to 300 µmol/L marginally decreased (*P* = 0.07) glycogen enrichment with ^14^C from [2-^14^C] sodium pyruvate.

With the exception of the interaction for CE (18:3) found in VLDL noted above, no evidence for interactions were observed (*P* > 0.10) between CC and DLM for any of the outcomes examined; therefore, main effects of CC and DLM are discussed.

## Discussion

In nonruminant species, the essential role of choline and Met to prevent lipidosis has been demonstrated and feeding choline- and Met-deficient diets is a common methodology to induce hepatic steatosis and lipidosis^5,6^. Deficiencies in these lipotropic nutrients may also contribute to lipidosis in ruminants^3^. Given choline and Met have the biochemical ability to directly or indirectly support synthesis of PtdChol^12^, a required component of VLDL, both nutrients have the potential to support lipid export in ruminants, although the mechanism to prevent lipidosis in dairy cows is ambiguous. Export of TG in VLDL did not appear to be affected by increasing DLM^10^, an observation that is consistent with unchanged cellular TG as DLM was supplied and increased in the present experiment and unchanged liver lipids in periparturient cows supplemented with rumen-protected forms for Met^13^. In addition, total PtdChol and PtdEth in VLDL were not affected by DLM supply (Table 1). Taken together, these data do not support a role for Met in TG export as VLDL to prevent lipidosis in bovine hepatocytes.

Feeding rumen-protected choline has either not affected, or decreased liver TG^7^. The presence of CC in culture media marginally decreased the accumulation of cellular TG in hepatocytes examined here (Fig. 1). As a direct precursor of PtdChol, CC can reduce cellular TG by increasing the export of FA as TG in VLDL in nonruminant species^14^. Although CC and DLM did not affect any individual species of PtdChol or total PtdChol species identified in VLDL, the negative correlation between total PtdChol and cellular TG (Table 1) support a role for increased PtdChol to limit lipid accumulation in hepatocytes. Using a similar primary bovine neonatal hepatocyte cell culture protocol, we previously reported increased VLDL export from hepatocytes as CC supply was increased using a bovine-specific dual-antibody ELISA technique that quantified VLDL particles^10^. In the current experiment, VLDL were isolated by SEC-FPLC before total TG in the VLDL fraction were quantified by colorimetric assay. The presence of total TG in VLDL was not altered by the presence of CC but was quadratically decreased by increasing CC supply (Fig. 1). Differences between these studies could be attributed to range of CC supply examined as concentration of CC reached 4528 μmol/L in the previous experiment^10^ while the current study focused on dose-titration with a lower maximal CC supply. Alternatively, differences in analytical methods may explain the increase in VLDL particles previously observed, but lack of increased total VLDL observed here as the size and number of VLDL particles are sensitive to choline deficiency^14,15^. Lack of correlation between cellular TG and VLDL TG quantified by colorimetric assay might suggest additional mechanisms for CC to prevent hepatic lipidosis.

In addition to promoting hepatic lipidosis, choline- and Met-deficient diets diminish mitochondrial function^11,16^ and ß-oxidation in rodents^17,18^. Given their apparent requirement to optimize oxidation^11,18^, the ability to modulate oxidative enzyme gene expression^8,19^, and the potential to alter substrate availability, supporting FA oxidation may be an alternative means of choline and Met to limit TG accumulation. The presence of CC in culture media increased the rate of conversion of [1-^14^C]C16:0 to CO_2_ and although the rate of conversion to ASP was not altered, BHB export was decreased. The misalignment of ASP and BHB export can likely be attributed to period of measurement, given that ASP was collected as a measure of ketone body production and a potential indicator of incomplete oxidation of [1-^14^C]C16:0 during the final 3 h of incubation after hepatocytes were exposed to an additional 1 mmol/L of FA. It is worth noting that despite increased oxidative capacity in the presence of CC, increasing CC supply marginally decreased complete oxidative capacity (Fig. 2), and could reflect a decrease in substrate availability due to decreased cellular TG, or decreased cellular TG stimulated capacity for complete oxidation of [1-^14^C]C16:0. Thus, the accumulation of TG under the influence of CC or DLM supply during the first 21 h of incubation likely influenced, and cannot be separated from, rates of oxidation during the final 3 h. Taken together, these data suggest that supplied choline increased the capacity for complete oxidation of FA. Increasing rates of complete oxidation are in alignment with decreased cellular TG in this and previous experiments^1,20^, although the mechanism remains unclear. Choline may have supported oxidative capacity by improving mitochondrial function^21^, preventing mitochondria injury from oxidative stress^11^, or decreasing TG accumulation. In other species, changes in FA oxidation have demonstrated links between these pathways and future research could further explore the role of choline in these hepatocellular functions by use of deuteromics or other metabolomic techniques^22^.

Within the current experimental design, we are unable to determine if decreased TG resulted from increased oxidation, if increased FA oxidation was enabled by decreased cellular TG, or if they were supported simultaneously. Furthermore, it is not yet clear how these improvements in lipid metabolism impacted other metabolic pathways. Interestingly, LPC 18:0, a choline-containing lipid that is derived from PtdChol by the activity of phospholipase A_2_^23^, was sensitive to CC supply and positively correlated to cellular TG (Table 1). LPC is considered a bioactive proinflammatory lipid that is associated with apoptosis and is known to affect the activity of enzymes controlling extracellular FA uptake by hepatocytes^23,24^. Increased cellular LPC promotes the protein expression of enzymes involved in FA uptake, while inhibition of phospholipase A_2_ decreased cellular LPC and cellular TG in HepG2 cells, an immortalized human hepatocyte cell line^23,24^. If LPC secreted within VLDL is an indicator that intracellular LPC concentration had been altered by CC treatment, it could support decreased cellular uptake of FA into hepatocytes and limited the availability of [1-^14^C]C16:0 for metabolism. Decreased LPC by CC, and the positive correlation between cellular TG and LPC, is of specific interest and should continue to be investigated.

The capacity for complete or incomplete oxidation of [1-^14^C]C16:0 did not appear to be sensitive to DLM supply under the conditions of this experiment. Increasing potential in vivo supply of Met by supplementing the Met analog D,L-2-hydroxy-4-(methylthio)-butanoic acid did not appear to affect the ex vivo capacity for complete oxidation of [1-^14^C]C16:0 in liver slices collected from postpartum dairy cows^25^. Similar to CC, the presence of DLM and increasing DLM supply decreased the export of BHB (Fig. 2); however, the ability of DLM to decrease BHB export did not appear to be due to limited substrate availability as the presence of DLM did not affect cellular TG. The ability of DLM to decrease BHB export without altering the capacity of complete or incomplete oxidation of [1-^14^C]C16:0 in the present experiment remains unclear, but may be related to experimental methodology as mentioned above.

Reactive oxygen species are a natural product of mitochondrial oxidative metabolism^26^ and unless neutralized, ROS can cause damaging oxidative stress and are considered a biomarker of oxidative stress in nonruminants^27^ and ruminants^28,29^. The imbalance between ROS production and neutralization by antioxidants^30^ can alter biological molecules and pathways, and provoke intracellular signaling cascades that induce proinflammatory cytokines^26^. In the present experiment, supplying CC to hepatocytes may have mitigated oxidative stress as accumulation of ROS in media was decreased. Reduced extracellular ROS is consistent with a similar experiment that reported a marginal decrease in ROS as CC supply was increased to primary bovine neonatal hepatocytes^10^. Oxidative stress and inflammation have been implicated in the pathogenesis of several liver diseases^30^ that can be induced by choline or Met deficiencies^11^. Choline infusion increased serum choline in calves and attenuated the clinical and inflammatory response and oxidative stress of induced endotoxemia, potentially by modulating the balance between oxidative stress and antioxidant capacity^31,32^. Additionally, recent work suggests that the NEFA-ROS-JNK/ERK-mediated mitochondrial signaling pathway may lead to NEFA-induced hepatocyte apoptosis^33^. Although connections between this signaling pathway and choline have not been noted in dairy cattle previously, it warrants future exploration. Methionine supply did not appear to affect ROS in the current or previous cell culture experiments^9,10^.

Choline’s ability to decrease ROS could result from decreased production, increased neutralization, or the combination of both. Increased capacity for [1-^14^C]C16:0 oxidation induced by CC suggests ROS was not reduced by decreased production, as mitochondrial FA oxidation is a primary producer of ROS^34^. Alternatively, CC may have improved ROS neutralization. Hepatocyte-derived glutathione is the most abundant mammalian nonenzymatic antioxidant^35^, and is considered an oxidative stress biomarker because of its marked ability to scavenge ROS^36^. Choline- and Met-deficient diets can decrease liver glutathione^11^ potentially by limiting cysteine, the rate-limiting substrate for glutathione synthesis^37^. In the present work, total glutathione (GSH and GSSG) was not affected (*P* > 0.15) by CC and was marginally decreased (*P* = 0.10) by the presence of DLM (data not shown). Because GSH is oxidized to GSSG when it scavenges ROS or free radicals, the ratio of the redox buffering system may be a more sensitive oxidative stress biomarker and has been used in vitro^38^ and in vivo^39^ in other species. Despite a decrease in extracellular ROS, increasing supply of CC only marginally decreased GSH and did not affect GSSG or the ratio of GSH to GSSG (Fig. 4). In contrast to the current work, a previous experiment that cultured primary bovine neonatal hepatocytes in lower concentrations of DLM reported an increase in total cellular glutathione when DLM was increased from 0 to 40 μmol/L^9^. Differences between experiments are likely related to differences in DLM supply. Interestingly, the absence of CC or DLM did not appear to limit GSH synthesis and could suggest that the other media components, such as cystine, were able to support GSH synthesis. Inconsistent patterns of change between ROS and GSH could suggest CC mitigated ROS through alternative enzymatic or nonenzymatic means. Betaine, a derivative of choline, has also demonstrated antioxidant activity^40^. Inconsistencies between ROS and GSH have also been reported in vivo when measured in transition cows^29,41^ and in vitro in cultured primary bovine neonatal hepatocytes^9^ and highlight that the relationship between extracellular ROS and intracellular GSH is unclear although potential roles for hydrogen peroxide and other oxidants as signaling molecules to mediate immune^42^ and inflammatory responses in paracrine and autocrine signaling have been demonstrated^6^. The consistent decrease in extracellular ROS with added CC in vitromay be a hepato-protective mechanism of choline that warrants continued study.

Previous cultures of bovine hepatocytes suggested that TG accumulation may limit capacity for gluconeogenesis^43^. Given choline’s apparent hepato-protective and lipotropic actions, the effect of choline and Met on cellular glycogen and glucose export was investigated. Depriving hepatocytes of CC decreased glycogen in the present experiment, and liver glycogen is similarly depleted in rodent models of non-alcoholic fatty liver disease or induced by choline deficiency^44^. As CC supply increased, glycogen increased (Fig. 5) and is consistent with in vitro^8^ and in vivo experiments that increased choline supply^45^. Liver glycogen and TG are negatively correlated in dairy cows^45^ and other species^46^ and the dose responsiveness of liver glycogen to increased choline supplementation could be related to the concomitant decrease in liver TG. The mechanism of CC to increase cellular glycogen in not clear but is not likely due to glucose uptake since bovine liver lacks glucokinase activity^47,48^. Given this, cellular glycogen reflects an increase in glyconeogenesis, the synthesis of glycogen from glucose-6-phosphate, derived from gluconeogenesis, rather than exogenous glucose^49^. Choline chloride may have shifted the fate of glucose-6-phosphate to glycogen and decreased glucose export in the culture system by molecular mechanisms requiring further exploration. In contrast to CC, DLM decreased glucose export without changing cellular glycogen (Fig. 5), suggesting a decrease in gluconeogenesis with increased DLM supply.

The contributions of gluconeogenic precursors shifts during the peripartum period^50^ and increased contribution of precursors that enter through pyruvate highlight the importance of pyruvate flux around the time of calving^51,52^. Pyruvate can be oxidized for energy, or pyruvate can be carboxylated to OAA by *pyruvate carboxylase* (EC 6.4.1.1) to increase oxidative capacity or be used for gluconeogenesis. We previously reported CC supply induced *pyruvate carboxylase* expression in a previous hepatocyte culture^8^, and we hypothesized that CC would increase the use of pyruvate for gluconeogenesis. The presence of CC marginally decreased the conversion of [2-^14^C]pyruvate to CO_2_, sparing potential gluconeogenic carbon from oxidation; however, conversion of [2-^14^C]pyruvate to glycogen did not appear to be affected by CC supply using the experimental design described here. Considering that glycogen was likely derived from gluconeogenesis, it could be inferred that [2-^14^C]pyruvate conversion to glucose was not sensitive to CC supply or hepatic lipidosis. Similarly, previous work suggested pyruvate and alanine conversion to glucose is not as sensitive to hepatic lipidosis as propionate, the main glucose precursor in ruminants^25,43,53^. Decreased enrichment of glycogen with pyruvate in the present experiment might imply a decrease in pyruvate conversion to glucose. Alternatively, the CC-induced increase in cellular glycogen may have been realized before the final 3 h incubation and [2-^14^C]pyruvate-glucose was diluted in the larger glycogen pool. Future methods that quantify the glycogen pool before substrate labeling would be able to further elucidate this. The unchanged rate of [2-^14^C]pyruvate conversion to glycogen combined with diluted glycogen enrichment with [2-^14^C]pyruvate suggests alternative glucogenic precursors supported gluconeogenesis and subsequently glyconeogenesis.

Several of the amino acids included in excess in the basal media are considered glucogenic and enter as different intermediates of the TCA cycle for gluconeogenesis. [2-^14^C]pyruvate spared from oxidation by increasing DLM supply did not appear to support gluconeogenesis as the conversion of [2-^14^C]pyruvate to glycogen was not affected (Fig. 6). Combined with the marginal decrease in glycogen enrichment with [2-^14^C]pyruvate as DLM supply was increased without a change in the glycogen pool by DLM could suggest increasing DLM supply limited the use of pyruvate for gluconeogenesis.

In agreement with our hypothesis, choline altered palmitate and pyruvate carbon flux through pathways of oxidation and glucose metabolism in primary bovine neonatal hepatocytes. The lack of interaction between CC and DLM for nearly all of the outcomes examined support unique biological roles for the methyl donors. Choline demonstrated hepato-protective affects by limiting ROS and decreasing cellular TG, while supporting complete oxidation of FA and glycogen storage, regardless of Met supply. Conversely, treatment with Met at these doses did not influence palmitate metabolism or lipid-related pathways. These findings support separate roles for choline and Met and are consistent with in vivo observations. Choline’s ability to alter carbon flux is of interest and future research should elucidate the mechanism of this regulation in hepatocytes.

## Methods

### Isolation and cell culture

Primary bovine neonatal hepatocytes were isolated from 4 Holstein bull calves < 7 d of age by collagenase perfusion of the caudate process as previously described^54^. The same 4 calves and hepatocytes from each calf were used for all of the experiments described below. Isolated cells were plated on 35-mm Eppendorf culture plates (Eppendorf, Hauppauge, NY) in Dulbecco’s Modified Eagle’s Medium (DMEM, 2902, Sigma-Aldrich, St. Louis, MO) supplemented with 20% fetal bovine serum (FBS) and 1% antibiotic, antimycotic solution (A5955, Sigma-Aldrich, St. Louis, MO). Four h after initial plating, media was refreshed with a 10% FBS and 1% antibiotic, antimycotic DMEM media. All animal use and handling protocols were approved by the University of Wisconsin-Madison College of Agricultural and Life Sciences Animal Care and Use Committee. All experiments were performed in accordance with relevant guidelines and regulations. Radioactivity work was conducted in accordance with the University of Wisconsin-Madison Environmental Health and Safety guidelines and regulations.

### Choline, methionine, and fatty acid treatments

Monolayer cultures of cells were maintained for 24 h and were at least 80% confluent before exposure to treatments. Cells were randomly assigned to increasing concentrations of choline chloride (CC) and d,l-methionine (DLM), in a factorial arrangement. To control the concentration of CC, DLM, and remove glucose, media was made in-house according to the formulation of DMEM 2902 (1.25 mmol/L sodium pyruvate; Sigma-Aldrich, St. Louis, MO) with L-Met and glucose omitted and fortified with a vitamin mixture that was made according to the formulation of the commercially available Basal Medium Eagle (BME) vitamin solution (6891; Sigma-Aldrich, St. Louis, MO) without CC. Basal culture media was the resulting glucose-, DLM-, and CC-free media containing standard concentrations of essential L-amino acids and 1% antibiotic, antimycotic. Volumes of sterile CC and DLM were added to cell culture plates containing basal media to achieve final treatment concentrations of 0, 10, 100, and 1000 μmol/L CC and 0, 100, and 300 μmol/L DLM in a factorial arrangement. Treatment concentrations were selected to represent a physiologically relevant dose-titration^10,55^ and test the absence and presence of CC or DLM. A FA cocktail was added to all treatments to achieve 1 mmol/L FA in media. Stocks of FA C14:0, C16:0, C18:0, C18:1n-9 *cis*, C18:2n-6 *cis*, and C18:3n-3 *cis* (Sigma-Aldrich, St. Louis, MO) were bound to bovine serum albumin (BSA; Merck Millipore, Billerica, MA) by the method previously described^56^ and combined in molar ratios to create a FA cocktail that mimicked the profile of circulating FA observed in dairy cows at the time of calving^57^. The FA cocktail was comprised of 3% C14:0, 27% C16:0, 23% C18:0, 31% C18:1n-6 *cis*, 8% C18:2n-6 *cis*, and 8% C18:3n-3 *cis.* All reagents used were of cell-culture grade or the highest purity available (Sigma-Aldrich, St. Louis, MO). All treatments were applied in triplicate and repeated in 4 independent preparations of cells. Within each cell preparation, treatments were replicated in 4 parallel incubations to allow for harvest of different outcomes: reactive oxygen species and glutathione (1), cellular TG and export of glucose, BHB, and VLDL (2), palmitate metabolism (3), and pyruvate metabolism and cellular glycogen (4).

### Reactive oxygen species and glutathione

After 24 h of treatment exposure, media was harvested from each plate and pooled within triplicate for immediate quantification of ROS by fluorometric assay (mol). After removal of media, cell lysates were collected from each plate in 0.5 mL cold 2-(*N*-morpholino)ethanesulphonic acid (MES) buffer (0.2 mol/L 2-(*N*-morpholino)ethanesulphonic acid, 0.05 mol/L phosphate, and 1 mmol/L EDTA, pH 6.0), pooled within triplicate, and homogenized. Cell homogenates were centrifuged at 10,000×*g* for 15 min at 4 °C before the supernatant was removed and an equal volume of metaphosphoric acid (1.25 mol/L) was added to the supernatant. Samples were then vortexed and centrifuged at 2500×*g* for 2 min before the supernatant was collected and stored at − 20 °C for subsequent quantification of glutathione (GSH) and glutathione disulfide (GSSG) by colorimetric assay (Glutathione Assay Kit, Cayman Chemical, Ann Arbor, MI). According to the manufacturer’s protocol, both GSH and GSSG were measured to reflect total glutathione. An aliquot of sample was used to derivatize GSH with 2-vinylpyridine before measuring GSSG separately. Measured GSSG was then subtracted from total glutathione to calculate GSH by difference.

### Cellular triglyceride, glucose and BHB export, and VLDL lipidomics

After 24 h of treatment exposure, media was harvested from each plate, pooled within triplicate, and stored at − 80 °C for subsequent quantification of glucose (Autokit Glucose, Wako Diagnostics, Richmond, VA) and BHB by colorimetric assay using a commercial kit (Catachem Inc., Bridgeport, CT) utilizing an autoanalyzer (CataChemWell-T, Awareness Technologies, Westport, CT). An aliquot of media was fractionated by size exclusion chromatography by fast protein liquid chromatography (SEC-FPLC), as described previously^58^, to isolate TG-rich lipoproteins. The fraction corresponding to TG-rich lipoproteins was collected and an aliquot used to quantify total VLDL TG by colorimetric assay (Pointe Scientific, Canton, MI). From the same fraction, untargeted lipidomics was performed using TripleTOF 5600 mass spectrometer (AB Sciex, Concord, Canada) as previously described^59^. The hepatocyte isolation and culture system are void of intestinal chylomicron and their TG; therefore, this fraction was considered to represent VLDL.

After removal of media, cells were rinsed with 1 mL of Ca-free Krebs buffer and individual plates were stored at − 20 °C with 1 mL dissociation buffer (2.68 mmol/L KCl, 1.47 mmol/L KH_2_PO_4_, 0.137 mol/L NaCl, 8.06 mmol/L Na_2_HPO_4_, 1.0 mmol/L Na_2_ EDTA-2H_2_O, pH 7.4) for subsequent quantification of cellular TG and DNA. Plates were thawed on ice and cells from each plate were collected into microcentrifuge tubes and sonicated in 2 mL dissociation buffer. A 750 µL aliquot was removed and lipids extracted according to the method of Folch et al^60^***.*** Lipids were resuspended in 300 µL of 1% Triton X-100 CHCl_3_ and analyzed for TG by colorimetric assay (L-Type Triglyceride M, Wako Diagnostics, Richmond, VA). From the remaining sample, an aliquot was removed for total DNA quantification by Hoechst assay based on the method of Labarca and Paigen^61^ using a calf thymus DNA standard. Total cellular TG and VLDL TG was normalized to corresponding total DNA within each culture plate prior to averaging within triplicates. Total glucose and total BHB in the pooled media sample were normalized to total DNA averaged across the triplicate. Due to technical difficulties, data from one cell preparation was removed from the cellular TG, VLDL TG, lipidomics, and glucose and BHB export data; therefore, statistical analysis of those outcomes was performed with data from 3 cell preparations.

### Measuring metabolic flux

#### Palmitate oxidation

Following treatment exposure for 21 h, media was replaced with a treatment media that omitted the 1 mmol/L FA cocktail and cells were incubated with [1-^14^C]C16:0 for 3 h before CO_2_ and acid-soluble products (ASP), including ketone bodies, were collected as previously described^62^. Briefly, treatment media was removed and each plate was placed into a straight-side wide-mouth 60 mL jar (Nalgene, Thermo Scientific, Waltham, MA). Treatment media was added to each plate and BSA-bound C16:0 containing [1-^14^C]C16:0 (Perkin Elmer, Waltham, MA) was added to achieve a final concentration of 1 mmol/L C16:0 with approximately 1,000,000 disintegrations per minute (DPM) per plate. Media was gassed with 95% O_2_, 5% CO_2_ and jars were immediately sealed with lids modified to hold rubber stoppers (Kimble Chase, Vineland, NJ) fitted with a hanging plastic center well (Kimble Chase, Vineland, NJ) containing a filter paper strip. Jars were incubated at 37 °C for 3 h before incubations were moved to room temperature and terminated by injection of 0.2 mL of 5 N HClO_4_ into media through the rubber stopper of the jar and CO_2_ was trapped by phenethylamine (0.2 mL, 100%) addition to each hanging center well containing the filter paper strip. After 1 h, center wells, including the filter paper strip and phenethylamine, were removed and placed in scintillation vials (Research Products International, Mt. Prospect, IL) and 5 mL of scintillation fluid (EcoLite, MP Biomedical LLC, Solon, OH) was added. To collect ASP, media was collected from plates into 15 mL conical tubes and plates were rinsed twice with 1 mL of 1X PBS that was added to conical tubes before samples were centrifuged for 10 min at 700×*g*. The supernatant was moved to a glass scintillation vial (DWK, Life Sciences, Millville, NJ), neutralized with 3 mol/L KOH, and 15 mL of scintillation fluid was added. Scintillation vials were sealed, mixed, and stored overnight in the dark before radioactivity was determined by liquid scintillation counting. Two additional triplicates of cells treated with basal media were incubated to determine background radioactivity in CO_2_ and ASP. One triplicate was immediately terminated after the addition of [1-^14^C]C16:0 and the other was incubated in the absence of [1-^14^C]C16:0 and terminated after the 3 h incubation.

After removal of center wells and media, 1 mL of dissociation buffer was added to each plate and plates stored at − 20 °C for subsequent analysis of total DNA as previously described^61^. The rate of complete oxidation of palmitate was determined for each plate and expressed as pmol ^14^C substrate metabolized to ^14^CO_2_·µg DNA^−1^·h^−1^. The rate of incomplete oxidation of palmitate for each plate was determined as the synthesis of ASP over time and expressed as pmol ^14^C substrate incorporated into ASP·µg DNA^−1^·h^−1^. The rate determined for each plate was averaged across each triplicate.

#### Pyruvate metabolism

Following treatment exposure for 21 h, media was replaced with a treatment media that omitted the 1 mmol/L FA cocktail but contained 1% BSA, and cells were incubated in the presence of [2-^14^C] sodium pyruvate at approximately 275,000 DPM per plate for 3 h before CO_2_ was collected as described above. The addition of [2-^14^C] sodium pyruvate to media did not increase sodium pyruvate (1.25 mmol/L sodium pyruvate) already present in treatment media by more than 1%. After CO_2_ collection as described above, media was removed and cells were rinsed twice with 1 mL of 1X PBS before individual plates were stored at -20 °C with 1 mL dissociation buffer for subsequent analysis of cellular glycogen and total DNA as previously described^61^.

Cellular glycogen was precipitated based on the method of Lo et al^63^. Plates were thawed on ice and cells collected into microcentrifuge tubes and sonicated in 2 mL dissociation buffer. A 1 mL aliquot was moved to a new tube and 1 mL 60% KOH saturated with sodium sulfate was added and the sample was placed in a 90 °C dry heat block for 30 min. Samples were cooled and 3 mL of 100% ethanol was added and glycogen precipitated overnight at 4 °C. Glycogen was pelleted at 1800×*g* for 15 min at 4 °C and the supernatant discarded before the glycogen pellet was washed with 1 mL of cold 100% ethanol and pelleted again at 1800×*g* for 5 min at 4 °C. Isolated glycogen was then converted to glucose units by acid-heat hydrolysis^64^. The pellet was dried and dissolved in 1X PBS and moved to a cryogenic tube where an equal volume of 4 mol/L HCl was added and the sample placed in a 95 °C dry heat block for 2 h. The sample was cooled and neutralized with 10 mol/L NaOH before glucose was quantified by colorimetric assay (Autokit Glucose, Wako Diagnostics, Richmond, VA). A 50 µL aliquot of sample was moved to a scintillation vial and 5 mL of scintillation cocktail was added. Scintillation vials were sealed, mixed, and stored overnight in the dark before radioactivity was determined by liquid scintillation counting. Two additional triplicates of cells treated with basal media containing 1% BSA were incubated to determine background radioactivity in CO_2_ and glycogen. One triplicate was immediately terminated after the addition of [2-^14^C] sodium pyruvate and the other was incubated in the absence of [2-^14^C] sodium pyruvate and terminated after the 3 h incubation.

The rate of pyruvate oxidation was determined for each plate and expressed as pmol ^14^C substrate metabolized to ^14^CO_2_·µg DNA^−1^·h^−1^. The rate of pyruvate incorporation into glycogen was determined for each plate and expressed as pmol ^14^C substrate recovered in glucose·µg DNA^−1^·h^−1^. The enrichment of pyruvate in glycogen was calculated for each plate as total pmol of ^14^C substrate recovered in glucose divided by total pmol of glucose in glycogen. Rate and enrichment determined for each plate was averaged across each triplicate.

### Statistical analysis

In order to have normalized comparisons of treatments across cell preparations, ROS, glutathione, cellular TG, VLDL TG, cellular glycogen, glucose in media, rate of [1-^14^C]C16:0 oxidation, rate of [2-^14^C] sodium pyruvate oxidation, rate of [2-^14^C] sodium pyruvate incorporation into glycogen, and glycogen ^14^C enrichment were expressed relative to the 0 μmol/L DLM, 0 μmol/L CC, 1 mmol/L FA treatment within each independent cell preparation before statistical analysis. Data were analyzed by PROC MIXED of SAS 9.4 (SAS Institute Inc., Cary NC) in a model that accounted for fixed effects of CC, DLM, the interaction of CC and DLM, and the random effect of cell preparation. Contrasts were evaluated for CC that tested the effect of absence versus presence of CC (0 μmol/L vs. 10, 100, 1000 μmol/L), and linear and quadratic effects of CC when CC was present (10, 100, 1000 μmol/L). Contrasts were evaluated for DLM that tested the effect of absence versus presence of DLM (0 μmol/L vs. 100, 300 μmol/L), and 100 versus 300 μmol/L DLM. Contrasts were considered significant when *P* ≤ 0.05 and marginally significant when 0.05 < *P* ≤ 0.10. Data are reported as least-squares mean and standard error of the mean.

Individual lipids identified by untargeted lipidomics were normalized to cellular DNA and summed within lipid species class. Partial Pearson correlation coefficients between cellular TG and VLDL TG, as well as cellular TG and classes of lipid species identified in VLDL, were calculated in PROC CORR after normalized data were centered within cell preparation. Because of the role of choline and Met in PtdChol and PtdEth synthesis and their importance to VLDL synthesis, individual lipid species of PtdChol and PtdEth were investigated further. Normalized values of individual PtdChol and PtdEth lipids as well as total lipid species were log transformed and expressed relative to the 0 μmol/L DLM, 0 μmol/L CC, 1 mmol/L FA treatment within each independent cell preparation before effects of CC and DLM described above were investigated. *P* values for the effects of CC and DLM on individual species of PtdChol and PtdEth were adjusted using the BH method^65^ to control the False Discovery Rate (FDR) at 10%.

## Data Availability

The data that support the findings of this study are available, on reasonable request, from the corresponding author.
