# Author Correction: Palmitate and pyruvate carbon flux in response to choline and methionine in bovine neonatal hepatocytes

**DOI:** 10.1038/s41598-021-86713-1

**Published:** 2021-04-07

**Authors:** T. L. Chandler, S. J. Erb, William A. Myers, Pragney Deme, Norman J. Haughey, J. W. McFadden, H. M. White

**Affiliations:** 1 Department of Dairy Science, University of Wisconsin-Madison, Madison, WI 53706 USA; 2 Department of Animal Science, Cornell University, Ithaca, NY 14853 USA; 3 Division of Neuroimmunology and Neurological Infections, Department of Neurology, Johns Hopkins University School of Medicine, Baltimore, MD 21287 USA; 4 Present Address: Department of Population Medicine and Diagnostic Sciences, College of Veterinary Medicine, Cornell University, Ithaca, NY 14853 USA

Correction to: *Scientific Reports* https://doi.org/10.1038/s41598-020-75956-z, published online 05 November 2020

William A. Myers, Pragney Deme and Norman J. Haughey were omitted from the author list in the original version of this Article. This has been corrected in the PDF and HTML versions of the Article.

The Author Contributions section now reads:

H.M.W. and T.L.C. designed the study. H.M.W. and J.W.M. acquired funding. T.L.C. and S.J.E. conducted the experiments. T.L.C., W.A.M., and P.D. conducted sample analysis and analyzed the data, T.L.C. and H.M.W. interpreted the results, and drafted the manuscript. All authors reviewed the manuscript and approved the final version to be published.

The Acknowledgements section now reads:

This research was supported by funding provided through the Real Science Initiative Grant by Balchem Corporation (New Hampton, NY), The New York State Centers for Advanced Technology, and National Institute of Food and Agriculture, U.S. Department of Agriculture, Award Number 2016-67015-24573. Gratitude is extended to Sandra J. Bertics (University of Wisconsin-Madison) for her technical assistance.

